# Muscle Cramps in Outpatients with Liver Diseases in Tokyo, Japan

**DOI:** 10.3390/medicina59091506

**Published:** 2023-08-22

**Authors:** Tatsuo Kanda, Reina Sasaki-Tanaka, Naoki Matsumoto, Shuhei Arima, Shini Kanezawa, Masayuki Honda, Mai Totsuka, Tomotaka Ishii, Ryota Masuzaki, Masahiro Ogawa, Hiroaki Yamagami, Hirofumi Kogure

**Affiliations:** Division of Gastroenterology and Hepatology, Department of Medicine, Nihon University School of Medicine, 30-1 Oyaguchi-kamicho, Itabashi-ku, Tokyo 173-8610, Japan; sasaki.reina@nihon-u.ac.jp (R.S.-T.); matsumoto.naoki@nihon-u.ac.jp (N.M.); arima.shuhei73@nihon-u.ac.jp (S.A.); kanezawa.shini@nihon-u.ac.jp (S.K.); honda.masayuki@nihon-u.ac.jp (M.H.); totsuka.mai@nihon-u.ac.jp (M.T.); ishii.tomotaka@nihon-u.ac.jp (T.I.); masuzaki.ryota@nihon-u.ac.jp (R.M.); ogawa.masahiro@nihon-u.ac.jp (M.O.); yamagami.hiroaki@nihon-u.ac.jp (H.Y.); kogure.hirofumi@nihon-u.ac.jp (H.K.)

**Keywords:** leg cramps, various liver diseases, VAS, levocarnitine, Shakuyaku-kanzo-to, diabetes mellitus

## Abstract

*Background and Objectives:* Muscle cramps are often observed in patients with liver diseases, especially advanced liver fibrosis. The exact prevalence of muscle cramps in outpatients with liver diseases in Japan is unknown. *Patients and Methods:* This study examined the prevalence of, and therapies for, muscle cramps in outpatients with liver diseases in Tokyo, Japan. A total of 238 outpatients with liver diseases were retrospectively examined. We investigated whether they had muscle cramps using a visual analog scale (VAS) (from 0, none, to 10, strongest), and also investigated their therapies. *Results:* Muscle cramps were observed in 34 outpatients with liver diseases (14.3%); their mean VAS score was 5.53. A multivariate analysis demonstrated that older age (equal to or older than 66 years) was the only significant factor as-sociated with muscle cramps. The prevalence of muscle cramps among patients with liver diseases seemed not to be higher. The problem was that only 11 (32.4%) of 34 outpatients received therapy for their muscle cramps. *Conclusions:* Only age is related to muscle cramps, which is rather weak, and it is possible that this common symptom may not be limited to liver disease patients.

## 1. Introduction

Muscle cramps are often observed in patients with cirrhosis; so-called “leg cramps”, characterized by severe pain in the calf muscles, occur several times a week and last for a few minutes, mainly while patients are at rest and/or during sleep [1].

Caused by dehydration or overexertion, muscle cramps as “exercise-associated muscle cramps” can be observed in healthy people [2]. Muscle cramps are also observed in patients undergoing hemodialysis or with type 2 diabetes [3].

The most frequently reported symptoms from patients with end-stage liver disease are pain, breathlessness, muscle cramps, sleep disturbance, and psychological symptoms [4]. Muscle cramps were prevalent in ~65% of cirrhotic patients [4,5], and were associated with lower serum albumin and a diminished quality of life in patients with cirrhosis [5].

It has been reported that the muscle cramps experienced by ~64% of patients with cirrhosis can be caused by an overlap with alcohol use disorder (~45%), nonalcoholic fatty liver diseases (NAFLD) (26%), and hepatitis C (41%) in the United States [6]. Miwa et al. reported that some of the 24 (64.9%) of 37 patients with cirrhosis studied in Japan who had muscle cramps also experienced alcohol use disorder (36%), hepatitis B (5%), and hepatitis C (19%) [7]. Thus, muscle cramps are observed as frequent disorders in patients with advanced liver fibrosis or cirrhosis [8].

However, the exact prevalence of muscle cramps in patients with liver disease in Japan, including patients with mild or moderate fibrosis, is unknown. So, we examined the prevalence of, and therapies for, muscle cramps in outpatients with liver diseases in Tokyo, Japan.

## 2. Patients and Methods

### 2.1. Study Design and Patients

This retrospective study enrolled 238 consecutive outpatients with liver diseases of any etiology and assessed their muscle cramps at the Division of Gastroenterology and Hepatology, Department of Medicine, Nihon University Itabashi Hospital, from 1 January 2020 to 31 December 2021. Patients were 20 years of age and older. The protocol of this single-center study followed the Declaration of Helsinki. The ethics committee of Nihon University Itabashi Hospital approved this retrospective study (protocol number RK-180911-12, which was approved on 11 September 2018 and 10 August 2022). Information regarding participation in this study was posted on our institution’s website and informed consent was obtained from all patients. The diagnoses of the liver diseases were made by care physicians of the outpatient clinic of the Division of Gastroenterology and Hepatology, Department of Medicine, Nihon University Itabashi Hospital.

### 2.2. Evaluation of Muscle Cramps

We asked participants whether they experienced muscle cramps or not using a visual analog scale (VAS) (from 0, none, to 10, strongest) and whether they received therapies for their muscle cramps. We recorded the responses using electronic medical records. We retrospectively double-checked the responses, treatments received, and other parameters.

### 2.3. Serum Biochemical Tests and Hematological Tests

Serum biochemical tests, including liver function tests (aspartate aminotransferase (AST), alanine aminotransferase (ALT), γ-glutamyl transpeptidase (γ-GTP), total bilirubin, (albumin), and hematological tests, were performed according to standard methods [9]. A diagnosis of hepatitis B virus (HBV) or hepatitis C virus (HCV) infection was indicated by the positivity of hepatitis B surface antigens (HBsAgs) or anti-HCV antibodies, respectively. Antimitochondrial M2 antibodies or antinuclear antibodies were also measured for the diagnosis of primary biliary cholangitis or autoimmune hepatitis, respectively [10].

### 2.4. Definition of Cirrhosis

An ultrasonography was performed for all patients in the present study. Cirrhosis was diagnosed using a liver biopsy and transient elastography (FibroScan greater than 12 kPa) and/or signatures of cirrhosis were identified using ultrasonography, computed tomography (CT), and/or magnetic resonance imaging (MRI). The existence of varices in the esophagus and/or stomach on an upper gastrointestinal endoscopy also indicated the signs of cirrhosis [11]. The existence of ascites was evaluated by the imaging modalities, such as ultrasonography, CT, and/or MRI.

### 2.5. Statistical Analysis

Data are expressed as mean ± standard deviation (SD). Differences were evaluated using the Student’s *t* test or chi-square test with the Yates correction. A *p* value of <0.05 was considered to indicate a statistically significant difference. Items with *p* < 0.05 at the univariate analysis were retained for a multivariate logistic-regression analysis. For all tests, two-sided *p* values were calculated, and the results were considered statistically significant at *p* < 0.05. The statistical analysis was performed using DA Stats software (O. Nagata, Nifty Serve: PAF01644) and the Excel Statistics program for Windows 2010 (SSRI, Tokyo, Japan).

## 3. Results

### 3.1. Patient Characteristics

Muscle cramps were observed in 34 (14.3%) of 238 outpatients with liver diseases (Table 1). Of interest, there were no significant differences in the liver function tests between patients with and without muscle cramps. Although diabetes mellitus was observed in 48 (20.3%) of 238 patients, there were no differences regarding diabetes mellitus between patients with and without muscle cramps. Of 238 patients, 11, 5, and 0 patients had esophageal varices, ascites, or hepatic encephalopathy, respectively; i.e., most of this study’s patients had compensated liver function.

Only 21 (8.8%) of 238 patients had liver cirrhosis; 5 (14.7%) of 34 patients with muscle cramps and 16 (7.8%) of 204 patients without muscle cramps were cirrhotic patients. Among 14 patients with esophageal varices and/or ascites, 3 patients (21.4%) had muscle cramps: 1 patient cirrhotic due to nonalcoholic steatohepatitis (NASH), 1 patient cirrhotic due to HBV infection, and 1 patient cirrhotic due to alcohol-associated liver disease (ALD) had VAS-8, VAS-2, and VAS-3 muscle cramps, respectively.

A univariate analysis showed that the age of patients with muscle cramps was significantly higher than the age of patients without muscle cramps. A multivariate analysis also demonstrated that older age (equal to or greater than 66 years) was the only factor associated with muscle cramps (Table 2). Thus, aging is an important factor when it comes to muscle cramps in patients with liver disease.

### 3.2. Prevalence of Muscle Cramps in Various Liver Diseases

In this study, 58, 38, 3, 20, 44, 11, 17, 5, and 42 patients had HBV-related liver diseases, HCV-related liver diseases, coinfection with HBV and HCV, alcoholic liver diseases (ALD), nonalcoholic liver diseases (NAFLD), autoimmune hepatitis (AIH), primary biliary cholangitis (PBC), drug-induced liver injury (DILI), and other liver diseases, respectively (Table 3).

The univariate analysis showed that all three patients coinfected with HBV and HCV had muscle cramps (*p* = 0.000583), but only one (2.4%) of 42 patients with other liver diseases had muscle cramps (*p* = 0.0288). However, the size of the groups was too small to reveal reliable differences because both the HBV and HCV group included only three cases.

Notably, the mean VAS score was 5.53 ± 3.05, which indicates that patients with liver disease who experienced muscle cramps seemed to suffer severe pain. Patients with higher VAS scores of 8, 9, and 10 included those with various liver diseases. Three patients coinfected with HBV and HCV indicated VAS scores of 1, 2, and 7, respectively.

### 3.3. Treatment for Muscle Cramps in Outpatients with Various Liver Diseases

Next, we investigated the types of muscle cramp treatments for liver disease patients in Japan. Only 11 (32.4%) of 34 patients received therapy for their muscle cramps (nine and two patients took oral levocarnitine [12,13] and the Japanese traditional herbal medicine Shakuyaku-kanzo-to [14], respectively) (Table 4). Surprisingly, four patients who had the most painful muscle cramps (with a VAS score of 10) were not treated. Thus, several patients with muscle cramps and liver diseases were left untreated.

## 4. Discussion

We investigated the prevalence of muscle cramps in patients with liver disease, including patients with mild or moderate fibrosis, in Japan. We found that 34 (14.3%) of 238 outpatients with liver diseases had muscle cramps at a university hospital in Tokyo, Japan. We demonstrated that older age (equal to or more than 66 years) was the only factor associated with muscle cramps in patients with liver diseases. This prevalence may be smaller, as 37% of healthy people per year have muscle cramps as part of the spectrum of normal human physiology [15], although food and nutrition may affect the status of muscle cramps [16]. In this study, however, we observed that 67% of patients with muscle cramps had not yet received appropriate therapeutic options. Our study supports the previous report that the prevalence of muscle cramps was relatively high in chronic liver disease [17]. However, they reported that female sex, comorbid diabetes, and chronic kidney disease are related to muscle cramps in chronic liver disease.

A recent trend in the etiology of cirrhosis with or without hepatocellular carcinoma (HCC) is that the number of viral hepatitis-associated cirrhosis cases is decreasing in Japan and the United States [6,18,19], according to the progress of the treatment [20,21,22]. A nationwide survey of the etiology of liver cirrhosis in Japan revealed that HCV infection (48.2%) was the leading cause of cirrhosis in Japan, and HBV infection (11.5%) was the third cause of cirrhosis in Japan [18]. A recent nationwide survey also suggests that the decrease in viral hepatitis-related HCC, particularly HCV-associated HCC, highly contributed to the etiological changes, although viral hepatitis still is the main cause of HCC in Japan [19].

However, the number of ALD-cirrhosis and NASH-cirrhosis cases is still increasing. It is a major problem that patients with cirrhosis have symptoms including muscle cramps, pruritus, poor-quality sleep, and sexual dysfunction, although advance cirrhosis possesses symptoms of decompensation, including ascites, bleeding tendency, encephalopathy, infection, and/or jaundice [6]. Therapeutic options to combat these symptoms are also required.

There are several noninvasive methods for the diagnosis of fibrosis of the liver, which can be divided into two categories: morphological tests, such as elastography and serum biochemical tests, are established and are developing better than before, although a liver biopsy is the gold standard method for the staging of liver fibrosis [23]. Accordingly, more patients with milder liver diseases than cirrhosis are diagnosed than before. However, do patients with liver diseases and no cirrhosis have symptoms, such as muscle cramps? To find out, we investigated the prevalence of muscle cramps among the patients with liver disease in Japan.

Motor neuron disease, spinobulbar muscular atrophy, spinal muscular atrophy, radiculopathy, motor, and sensory peripheral neuropathies, peripheral nerve hyperexcitability, and neuromyotonia are neurogenic causes of symptomatic muscle cramps. Electrolyte imbalance, hepatic and renal dysfunction, vitamin deficiency, diabetes mellitus, (para)thyroid dysfunction, and chronic venous insufficiency, are non-neuromuscular causes of symptomatic muscle cramps [15]. If muscle cramps are observed in patients with liver diseases, we should rule out the diseases above.

It has been reported that painful muscle cramps are a recognized symptom of cirrhosis [1,5]. Based on their multivariate analysis, Iwasa et al. reported that factors associated with a muscle cramp frequency of daily to a few times per week in patients with chronic liver diseases (37.7% had cirrhosis) were female sex, HCV infection, and age [24]. They also reported that 25.1% and 36% of patients with muscle cramps experienced poor quality of life and sleep disturbance, respectively. In the present study, which included only 4.6% (11/238) of decompensated liver diseases, we did not demonstrate that HCV infection or female sex was a factor associated with muscle cramps using a univariate analysis (Table 1 and Table 3). In the present study, among patients with and without cirrhosis, respectively, 23.8% (5/21) and 13.4% (29/217) patients had muscle cramps (*p* = 0.9597), although this might be related to the small number of cirrhotic patients in our cohort. It is possible that patients without cirrhosis were suffering from painful muscle cramps.

Of interest, all three patients coinfected with HBV and HCV had leg cramps. In general, skeletal muscle cells could not support HBV or HCV replication. In human hepatocytes, HBV evades the induction of interferon and interferon-induced antiviral effects. HBV infection does not rescue HCV from the interferon-mediated response [25]. In an HBV and HCV coinfection model, direct viral interference is absent [26]. The establishment of an HBV infection and the HBV replication space is limited by the antiviral effects of type I interferon in a chronically HCV-infected liver [27]. In vitro HBV and HCV co-infection led to the decreased replication of both viruses [28]. Hepatitis viruses also activate the production of interferon and cytokines [29,30]. Why did we observe muscle cramps in patients coinfected with HBV and HCV? The mechanism of muscle cramps in HBV and HCV coinfected patients should be elucidated.

Muscle cramps were identified as nocturnal leg cramps in this study. Nocturnal leg cramps are a skeletal muscle disorder characterized by a suddenly occurring, painful, involuntary contraction of the calf, hamstrings, and/or foot muscles at night [31]. In the report from European countries, the estimated prevalence of muscle cramps among primary care patients aged 60 years and older is 46% [32]. One-third of these patients reported being woken up by cramps. The duration from onset was 5 years or more for about 60% [32]. According to the previous report [33], in the general population, muscle cramps were present in 40% of those over the age of 50, have an increased frequency with age, show no sex preference, and are associated with sleep disturbance and overall poor health. Of interest, in our study, an age of 66 years and older was the only factor associated with muscle cramps in patients with liver diseases.

In this study, we demonstrated that a small number (32.4%) of liver disease patients with muscle cramps received treatment for muscle cramps. Therapeutic options for muscle cramps should be required for patients with liver diseases. Even patients with severely painful muscle cramps were not treated with any medicine for muscle cramps. It is possible that these patients could not complain about their muscle cramps to their doctors, that their doctors did not notice their patients’ muscle cramps, or that their doctors could not readily recognize the painful muscle cramps and their need for treatment. This finding might be interesting for practical health care in Japan.

Although the exact mechanism of muscle cramps is still unknown, nerve function, energy metabolism, plasma volume, and electrolytes seem to be involved [34]. Muscle cramps in liver cirrhosis patients may have a neural component, as eperisone hydrochloride, which is known to inhibit mono- and multi-synaptic reflexes through an inhibitory action on α- and γ-efferent neurons in the spinal cord, was an effective treatment [35].

Muscle cramp frequency has been reported to be dramatically reduced by branched-chain amino acids (BCAAs) [36,37]. BCAAs have aliphatic side chains with a branch point, and comprise valine, leucine, and isoleucine [37]. In patients with cirrhosis, the nocturnal administration of BCAA granules could lead to a significant decrease in the occurrence of muscle cramps in the legs [38]. They also found that, after 3 months of the nocturnal administration of BCAA granules, the Fisher’s ratio significantly increased, compared to the daytime administration of BCAA granules, despite there being no significant difference in serum albumin levels or glycated albumin levels between these two groups [39]. BCAAs could improve albumin synthesis and insulin resistance, as well as the prevention of the occurrence of HCC and clinical decompensation in cirrhotic patients, resulting in an improvement in these patients [37]. BCAAs may inhibit muscle cramps through the possible improvement of the imbalance and consequent restoration of taurine production [36,37].

L-carnitine, known as levocarnitine, may be an important factor in glycogen synthesis and ATP production in rat hepatocytes [38]. Levocarnitine supplementation, with or without exercise, could improve muscle cramps in patients with liver cirrhosis [12,13,40]. Regarding the dose and duration of levocarnitine supplementation, a dose of more than 4200 mg/week and with a duration of at least 12 weeks seemed to prevent dialysis-related hypotension [41]. Most of the total body carnitine is found in skeletal muscle, with 2–3% in the liver and kidney [42]. Levocarnitine supplementation may be useful for the improvement of sarcopenia, muscle weakness, and/or muscle cramps and asthenia [43]. Serum carnitine concentrations were within standard levels in the majority of liver cirrhosis patients. In patients with concurrent hyperammonemia, however, levocarnitine supplementation reduced blood ammonia levels [44]. Further investigation was needed.

In Japan, Shakuyaku-kanzo-to, a traditional Japanese medicine, that is, Kampo medicine, has been used to treat muscle cramps in patients with liver cirrhosis when necessary [45]. If Kampo medicine contains many plant-derived chemicals having an ability to inhibit nerve action potential conduction, it may be possible that this medicine inhibits nerve conduction [46]. Muscle cramps frequently develop under abnormal serum K+ concentrations [14]. Shakuyaku-kanzo-to may normalize the intracellular and extracellular K+ balance [47]. Shakuyaku-kanzo-to also inhibits tetanic contraction in vivo [48]. In this study, several patients took levocarnitine or Shakuyaku-kanzo-to for their painful muscle cramps.

Taurine, orphenadrine, baclofen, pregabalin, zinc, and vitamin D are also safe, and can have beneficial effects on muscle cramps [49]. It was reported that taurine is a therapeutic agent for muscle cramps through the stabilization of the skeletal muscle cell membrane [50,51,52]. Taurin plays a role in osmoregulation, calcium transport, membrane stabilization, and anticonvulsant action [51]. Low-dose taurine was nontherapeutic, allowing other factors to influence cramp frequency, although a higher dose of 2 g of taurine/day improved the frequency of muscle cramps [51].

Orphenadrine, an anti-cholinergic drug, has been used to treat painful muscle spasms [53]. Orphenadrine plays a role in blocking sodium channels as an analgesic compound [54]. Orphenadrine is useful for the safe and effective treatment of muscle cramps in patients with cirrhosis [52]. Baclofendagger (Lioresal) is a derivative of gamma-aminobutyric acid and may be able to treat spasticity [55]. Muscle cramps are possibly related to peripheral nerve hyperexcitability. Pregabalin, a gamma-aminobutyric acid analogue, could reduce muscle cramps in patients with cirrhosis [56].

An association between serum zinc levels and muscle cramps has been reported, and zinc supplementation may lead to the improvement of muscle cramps in patients with cirrhosis [57]. Zinc compounds have anti-viral activity and affect mitogen-activated protein kinase (MAPK) signaling pathways [58,59,60]. MAPK activation may be associated with the generation of pain hypersensitivity [61]. Magnesium supplementation was reported to be effective for patients with liver cirrhosis, only some of whom suffered from muscle cramps [62,63]. It was reported that the median concentration of zinc in patients with chronic liver diseases was statistically lower than that in healthy control subjects [64]. Further studies will be needed.

**Figure 1:**
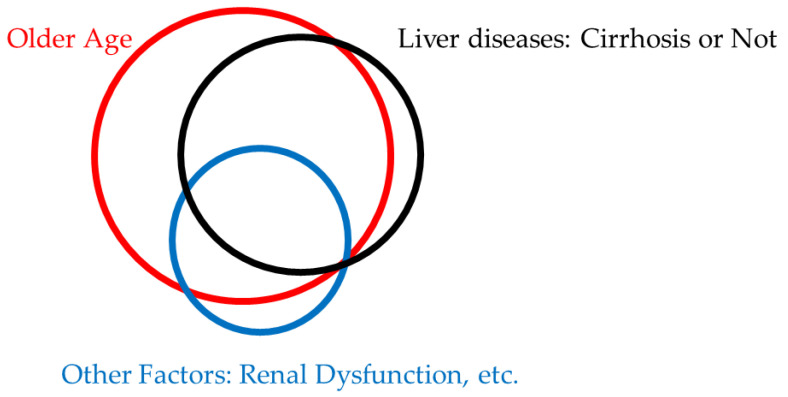
Potential reasons for muscle cramps in patients with various liver diseases.

Multiple risk factors may be associated with muscle cramps [65]. The potential reasons for muscle cramps seem to be various in patients with liver diseases (Figure 1 and Table 5). From our results, older age is also one of the potential reasons for muscle cramps in patients with various liver diseases.

It is reported that certain drugs are useful for the treatment of muscle cramps in patients with cirrhosis. The development and improvement of treatment options for painful muscle cramps in patients with or without cirrhosis is required. Representative causes and recommendations for prevention or therapeutic techniques for muscle cramps in patients with liver diseases are shown in Table 5.

Caffeine, alcohol, and illicit drugs are known to be exogeneous triggers of muscle cramps [15]. Among medications, lipid-lower medication (statins and fibrates), cardiovascular medication (diuretics, anti-arrhythmic medication, nifedipine, and amlodipine), antibiotics (cyclosporine and penicillin), lithium, cimetidine, methylphenidate, nicotinic acid, and others have been reported as exogeneous triggers of muscle cramps [15]. Statins activate the MAPK signaling pathway and can reduce the resting chloride channel conductance and lactate efflux in sarcoplasm, which are responsible for muscle cramps [66]. Alcohol consumption and drug–drug interaction exacerbate these phenomena.

The revised third edition of the clinical practice guidelines for liver cirrhosis, which was published in 2020 by the Japan Society of Hepatology [67], recommended that Shakuyaku-kanzo-to, levocarnitine, BCAAs, and zinc are proposed according to the pathological condition of muscle cramp-associated cirrhosis. However, in patients with cirrhosis with sarcopenia, addition of BCAA to exercise, dietary counselling and standard medical therapy did not improve muscle mass [68]. Of interest, AASLD practice guidance reported that muscle cramps are one of the symptoms of a deficiency of selenium [69]. EASL guidance recommended that albumin infusion or baclofen administration (10 mg/day, with a weekly increase of 10 mg/day up to 30 mg/day) are recommended in patients with muscle cramps [70]. The recommendations of the American Academy of Neurology, for example, mention only a few possible agents, and indicate that further studies are needed. As yet, it often remains a case of trial and error; we need more data about the effective drugs for muscle cramps [71].

EASL guidance also recommended that diuretics should be discontinued if incapacitating muscle cramps develop [70]. There are several retrospective studies that have excluded an association between diuretics and muscle cramps [72,73]. Although there is a report that muscle cramps were not a complication of diuretic use in patients with cirrhosis, despite the increasing frequency of cramps due to the use of diuretics in Child–Pugh B or C cirrhosis [8], diuretic therapy and medications are also important as an etiology of muscle cramps [74]. Among the antihypertensive agents, diuretics are most often associated with cramps [75]. The mechanism of diuretic-related cramping is likely associated with hypokalemia, hypomagnesemia, or volume contraction. Muscle cramps are often observed among hemodialysis patients [76]. We could not examine all medications in patients with or without muscle cramps in the present study. As older patients with liver diseases tend to be polypharmacy cases [77], careful attention should be paid to diuretic therapy and medications among patients with liver disease and muscle cramps. We do not completely exclude the possibility that high-risk medications and polypharmacy should modify the prevalence of muscle cramps among patients with liver diseases [78].

Recently, possible dysbiosis of microbiota and mitochondrial dysfunction were reported in hemodialysis patients with muscle cramps undergoing citrate dialysate treatment compared to patients without cramps [79]. Further studies will be needed to determine the association between microbiota and muscle cramps, although a relationship between several diseases and microbiota has been reported [80].

The number of patients in this study was relatively small. Although Cohen’s d values were added as a power analysis for the entire cohort (Table 1), the sample size of patients in subgroups seemed smaller. Therefore, we need to confirm our results in a larger prospective cohort. The main limitation of the study is that it has a retrospective design, which may significantly limit the results. A limitation of the present study is that we only used VAS to evaluate muscle cramps. The patients also answered and classified their cramps episodes retrospectively. This is associated with memory bias since patients might not clearly remember the intensity of their symptoms and could under- or overestimate it.

Questionnaires comprising multiple-choice questions [81], blood flow [82], and magnetic resonance imaging (MRI)-based imaging methods [83] have been reported as muscle cramps evaluation methods. Artificial intelligence-based systems may also detect muscle cramps among patients with liver diseases [84]. New evaluation methods for muscle cramps may be required.

Additionally, for the definition of cirrhosis we chose a relatively low elastography cut-off (12 kPa), which might include many individuals without cirrhosis and, thus, could have an impact on the conclusions of the study.

## 5. Conclusions

Although therapeutic options are required for muscle cramps, ~70% of patients with muscle cramps did not receive therapy for muscle cramps. Only age is related to muscle cramps, which is rather weak, and it is possible that this common symptom may not be limited to liver disease patients.

## 6. Addendum

We partly presented the content of this study at the 32nd Annual Conference of the Asian Pacific Association for the Study of the Liver, 15–19 February 2023, Taipei, Taiwan, and we also published the content in abstract-only form [85].

## Figures and Tables

**Table 1:** Characteristics of 238 outpatients with liver disease.

Items	All Patients (*n* = 238)	Muscle Cramps (+) (*n* = 34)	Muscle Cramps (−) (*n* = 204)	*p Values* ^1^	*Cohen’s d* ^1^
Age (years)	62.4 ± 13.9	67.8 ± 11.1	61.6 ± 14.2	*0.0161*	*0.486*
Gender (male/female)	111/127	17/17	94/110	*0.0570*	*N.A.*
Platelets (×10^4^/μL)	21.8 ± 11.4	20.5 ± 8.4	22.0 ± 11.9	*0.481*	*0.102*
AST (IU/L)	28.5 ± 24.4	27.0 ± 11.7	28.8 ± 26.0	*0.692*	*0.0631*
ALT (IU/L)	26.7 ± 26.1	24.3 ± 16.7	27.1 ± 27.4	*0.564*	*0.0872*
γ-GTP (IU/L)	60.4 ± 130.5	76.3 ± 155.4	57.7 ± 126.1	*0.909*	*0.0931*
Total bilirubin (mg/dL)	0.8 ± 0.4	0.7 ± 0.3	0.8 ± 0.4	*0.165*	*0.2*
Albumin (g/dL)	4.2 ± 0.4	4.1 ± 0.4	4.2 ± 0.4	*0.178*	*0.176*
Diabetes mellitus (+/−)	48/190	11/23	37/167	*0.0926*	*N.A.*
Skin pruritus (+/−)	51/187	11/23	40/164	*0.147*	*N.A.*

*n*, number; AST, aspartate aminotransferase; ALT, alanine aminotransferase; γ-GTP, γ-glutamyl transpeptidase; N.A., not available. ^1^ *p* values when comparing the muscle cramp (+) group with the muscle cramp (−) group.

**Table 2:** Factors associated with muscle cramps in patients with liver diseases.

Factor	Category	Odds Ratio	95% CI	*p Values*
Age (≥66 years)	(+/−)	2.500	1.135–5.503	*0.0228*

95% CI, 95% confidence interval.

**Table 3:** Muscle cramps in outpatients with various liver diseases.

Items	All Patients (*n* = 238)	Muscle Cramps (+) (*n* = 34)	Muscle Cramps (−) (*n* = 204)	*p Values* ^1^
HBV infection	58	5	53	*0.229*
HCV infection	38	3	35	*0.329*
HBV and HCV	3	3	0	*0.000583*
ALD	20	5	15	*0.273*
NAFLD	44	8	36	*0.562*
AIH	11	3	8	*0.413*
PBC	17	5	12	*0.136*
DILI	5	1	4	*0.782*
Others	42	1	41	*0.0288*
Total patients	238	34	204	N.A.

*n*, number; HBV, hepatitis B virus; HCV, hepatitis C virus; HBV and HCV, coinfection with HBV and HCV; ALD, alcohol-associated liver disease; NAFLD, nonalcoholic liver disease; AIH, autoimmune hepatitis; PBC, primary biliary cholangitis; DILI, drug-induced liver injury; N.A., not available. ^1^ *p* values when comparing the muscle cramp (+) group with the muscle cramp (−) group.

**Table 4:** Treatments for muscle cramps for liver disease patients.

VAS	N	Treatment, *N*
1	0	None
2	7	Levocarnitine, 1; None, 6
3	6	Shakuyaku-kanzo-to, 1; None, 5
4	0	None
5	9	Levocarnitine, 3; None, 6
6	0	None
7	1	Levocarnitine, 1
8	2	Shakuyaku-kanzo-to, 1; None, 1
9	2	Levocarnitine, 1; None, 1
10	7	Levocarnitine, 3; None, 4

VAS, visual analog scale; *N*, number.

**Table 5:** Representative causes and recommendations for prevention or therapeutic techniques for muscle cramps in patients with liver diseases.

Causes	Prevention or Therapeutic Techniques
Nerve dysfunction	Shakuyaku-kanzo-to, Orphenadrine, Baclofendagger, Pregabalin
Muscle atrophy	Taurine
Energy metabolism	Branched-chain amino acids, Levocarnitine, Zinc, Vitamin D
Plasma volume	Reduction in diuretics
Electrolytes	Shakuyaku-kanzo-to
Diabetes mellitus	Antidiabetic drugs

## Data Availability

All data underlying this article are available in this article.
